# Quantifying electron transfer reactions in biological systems: what interactions play the major role?

**DOI:** 10.1038/srep18446

**Published:** 2015-12-22

**Authors:** Emil Sjulstok, Jógvan Magnus Haugaard Olsen, Ilia A. Solov’yov

**Affiliations:** 1 Department of Physics, Chemistry and Pharmacy, University of Southern Denmark, DK-5230 Odense M, Denmark; 2 Laboratory of Computational Chemistry and Biochemistry, École Polytechnique Fédérale de Lausanne (EPFL), CH-1015 Lausanne, Switzerland

## Abstract

Various biological processes involve the conversion of energy into forms that are usable for chemical transformations and are quantum mechanical in nature. Such processes involve light absorption, excited electronic states formation, excitation energy transfer, electrons and protons tunnelling which for example occur in photosynthesis, cellular respiration, DNA repair, and possibly magnetic field sensing. Quantum biology uses computation to model biological interactions in light of quantum mechanical effects and has primarily developed over the past decade as a result of convergence between quantum physics and biology. In this paper we consider electron transfer in biological processes, from a theoretical view-point; namely in terms of quantum mechanical and semi-classical models. We systematically characterize the interactions between the moving electron and its biological environment to deduce the driving force for the electron transfer reaction and to establish those interactions that play the major role in propelling the electron. The suggested approach is seen as a general recipe to treat electron transfer events in biological systems computationally, and we utilize it to describe specifically the electron transfer reactions in *Arabidopsis thaliana* cryptochrome–a signaling photoreceptor protein that became attractive recently due to its possible function as a biological magnetoreceptor.

Electron transfer reactions have a vital importance in biological systems, being, for example, responsible for such acts as, activation of sensory proteins^1^, DNA UV-damage repair^2^, energy harvesting^3^, magnetic field sensing^4^,^5^ and many others. Three of these exemplary functions are illustrated in Fig. 1: the electron transfer reaction activates enzyme photolyase which then repairs a UV-damaged DNA^2^,^6^ Fig. 1A; a charge transfer processes through the cytochrome bc1 complex leads to formation of an electrostatic gradient through a membrane^7^,^8^,^9^, Fig. 1B; a light-triggered electron transfer induces activation of a photoreceptor protein cryptochrome^5^,^10^,^11^,^12^,^13^,^14^,^15^,^16^, Fig. 1C.

Even though the role of electron transfer reactions has been established in various biological systems^17^,^18^, it is difficult to observe such reactions experimentally under controlled conditions. In particular, experimental studies alone cannot describe electron transfers on the level of atomistic details, which, however, is often necessary for completing the interpretation of the underlying biophysical mechanisms. Alternatively, computational models of electron transfer processes provide reasonably robust approaches^14^,^16^,^19^,^20^ to characterize electron transfer reactions. It has been revealed^19^ that for a quantitative description of the electron transfer processes in a biological system, it is necessary to consider the entire system, and not just the electron donor and acceptor sites that are directly involved in the electron transfer process. This has been recently demonstrated for several different exemplary systems^19^,^21^, however, it remains largely unknown what interactions between the moving electron and the rest of the protein constitute the driving force for the electron transfer reaction. In the present investigation we have addressed this problem and used time dependent (TD) density functional theory (DFT) to describe electronic transitions in an exemplary biological system. In particular, we have considered the electron transfer in *Arabidopsis thaliana* cryptochrome (*AtCry*)^22^, a process that has been studied extensively both experimentally^23^,^24^,^25^,^26^,^27^ and computationally^10^,^11^,^13^,^14^,^16^,^19^,^28^,^29^ throughout the last decade.

Cryptochromes are flavoproteins, involved in light-dependent signaling pathways of several vital biological processes, such as the regulation of the hypocotyl growth in plants and entrainment of circadian rhythm in animals^30^. Cryptochromes were also proposed to act as sensors for the geomagnetic field and assists many animals in long-range navigation^5^,^10^,^11^,^13^,^14^,^16^,^28^,^31^.

The biological activation of cryptochrome arises from light-induced formation of a radical pair through electron transfer between a flavin cofactor (FAD) and a triad of tryptophan residues^30^, which constitute the active site of the protein. Figure 2A illustrates the process, by showing the three consecutive electron transfers between flavin and the tryptophans of the triad, W_A_, W_B_ and W_C_, which in the case of *AtCry* have the amino acid indices 400, 377 and 324, respectively. The three electron transfers are labeled ET1, ET2 and ET3, and occur after flavin photoexcitation, leading to formation of two intermediate radical pair states, 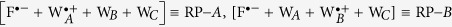 and the final, persistent, radical pair state 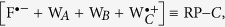
from the initial inactive *closed shell state*, 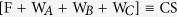. The interconversion of these four electronic states is governed by the free energy surfaces of the corresponding electronic states, and their crossings, as depicted schematically in Fig 3. Each electronic state is in a certain optimised configuration of the cryptochrome active site, which correspond to a minimum on the respective energy surface. In the present study we consider the so-called forward electron transfer reactions, ET1, ET2, ET3, that lead to fast cryptochrome activation, not the protonation or recombination reactions, that were also observed in cryptochrome, on longer timescales^24^,^30^,^32^ and are expected to stabilize the signaling state of the protein. Following earlier computations^19^, sequential electron transfer is expected to lower the energy of the radical pair states, and it is even possible that the persistent radical pair state RP-C becomes the ground state of the system, as shown schematically in Fig. 3 through the dashed black line, which describes the closed shell state.

In the present investigation, the impact of the molecular environment on the electron transfers in *AtCry* is quantified through the ‘vacuum model’ (Fig. 2A) where only the active site is considered and all the protein interactions are neglected, and the ‘environment model’ (Fig. 2B), where the complete protein structure and surrounding water molecules are taken into account. By enabling different contributions to the electrostatic interactions between the active site and the surrounding atoms in the environment model, we investigate which interactions turn out to be key in propelling the electron through the protein. We quantify the effect of different electrostatic and polarization interactions arising in the active site of *AtCry* and suggest a general workflow for treating, computationally, electron transfer reactions in biological systems.

## Results

### Active site interactions with the environment

One of the main impacts of the protein matrix and its surrounding on the electron transfer process in the active site is due to electrostatic interactions and polarization between the active site and the surrounding atoms. The interactions can be described through the multipole expansion series. Figure S2 illustrates the electronic excitation spectra in the active site of *AtCry*, where its interaction with the environment has been decomposed into five components representing: the partial charges (*q*_0_), dipole moments (*d*) and quadrupole moments (*Q*) ascribed to each individual atom of the protein, and induced dipole moments arising on each atom of the environment, calculated according to the ground state of the active site 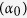, and induced dipole moments of all atoms of the environment 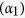 that take into account also the charge redistribution in the active site upon electronic excitation.

Alternatively, fitted point charges 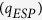 placed on each atom of the environment can be used to reproduce the electrostatic potential of the system, replacing the 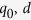, and *Q* terms introduced above.

Below we consider the importance of all five interactions (*q*_0_, *d*, *Q*, *α*_0_, *α*_1_) and deduce those that play the major role on electron transfers in *AtCry*.

### Electron transfer driven by polarization

The impact of electrostatic interactions and polarization on the *AtCry* active site electronic excitation spectra can be quantified by evaluating the strength of each interaction. Figure 4 shows how the excitation energies in different structural configurations of cryptochrome active site are changed upon excluding different electrostatic and polarization contributions from the environment.

In particular the figure illustrates the change in the excitation energies of the different electronic states in cryptochrome relative to the case where all static (*q*_0_, *d*, *Q*) and polarization (*α*_0_, *α*_1_) interactions are accounted for.

Polarization of the environment atoms arising due to electronic excitations in the active site (*α*_1_) seem to have a negligible impact on the electronic spectra of *AtCry* active site, as follows from Fig. 4 column 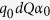, meaning that physically the differential environment polarization contributions of the electronic states RP-A, RP-B, RP-C are of little importance for the electron transfer reactions, at least in *AtCry*. However, neglecting the polarization of the environment atoms completely, i.e., representing the environment only through electrostatic interactions, raises the excitation energies for all of the structural configurations of the *AtCry* active site, see Fig. 4 column labeled 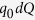. Thus in the case of the CS_opt_ configuration of the active site, the energy of RP-A, is 0.62 eV higher, while the energies of the radical pair state RP-B and RP-C are overestimated by 0.9 eV and 1.3 eV, respectively. For the optimized structural configuration RP-A_opt_ the deviations in the excitation energies are smaller, being; 0.26 eV, 0.45 eV and 0.95 eV for the RP-A, RP-B and RP-C states respectively; for the optimized structural configuration RP-B_opt_, the energies of the RP-A and RP-B states are overestimated by just 0.06 eV and 0.01 eV respectively, while RP-C is overestimated by 0.55 eV; finally, for the structural configuration RP-C_opt_, the errors of the RP-A excitation is overestimated by 0.14 eV, while for the RP-B and RP-C excitations, the energies deviate by 0.01 eV and 0.003 eV respectively. Although some excitation energies match, the performed analyss shows clearly that it is impossible to describe all the electronic excitations in the four structural states of *AtCry* active site simultaneously with a reasonable accuracy, once the polarisation term is neglected.

Excluding further electrostatic interactions from the molecular environment, i.e. representing the environment through, point charges and dipole moments or point charges only, see Fig. S2 and 4 columns labeled *q*_0_ and *q*_0_*d*, leads to further shifts in the energy of the excited states. Thus, the maximal deviation of energy in *AtCry* active site electronic spectra were the environment is represented through either charges and dipole moments or charges only, is 0.73 eV and 0.84 eV respectively, as compared to a model where also quadrupole and polarization interactions are included.

It should be noted that representing the environment through point charges and dipole moments can lead to inconsistent results, due to a inconsistent convergence of the multipole expansion series method used here^33^. Therefore, usually charges, dipole moments and quadrupole moments are required to get a reasonable convergence of the multipole expansion.

### Alternative description of electrostatic interactions through electrostatic potential fitting

An alternative approach to describe electrostatic 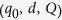 interactions, could be achieved through fitting the point charges of all atoms to reproduce the electrostatic potential of the system^34^. Although this approach can be equivalent to the multipole expansion scheme, some differences arise. When describing the electrostatic potential with the fitted charges, the electronic excitation spectra for all structural configurations of *AtCry* active site are energetically underestimated compared to the corresponding spectra obtained through the multipole expansion. The largest deviation is about 0.37 eV, indicating that although physically both methods are similar, it is not possible to reproduce as accurately the electrostatic properties of a molecular system through only charges.

Combining the ESP charges with the polarization contributions to the electrostatic potential, calculated for the closed shell state of the active site changes the electronic excitation spectrum as seen in Fig. S2, (column 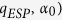, by shifting the energy levels closer to the analagous 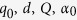 case. The energies, however, turn out to be systematically underestimated in all the electronic states, by not more than 0.2 eV (see Fig. 4), There is no simple physical explanation for the systematic underestimation of electronic states, and the observed effect is attributed to the differences in the two methods used.

### Energetics of the electron transfer

Figure 5A illustrates the potential energy surfaces for the key electronic states (CS, RP-A, RP-B and RP-C) of the active site of *AtCry*, where the environment is represented by the ESP-fitted charges and induced dipoles 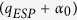. The calculated energies appearing in the diagram (symbols), have been computed relatively to the energy of the CS state, optimized in the CS configuration of the active site, i.e. CS_opt_. The calculated total energies for the electronic states in the four configurations, are compiled in Table 1.

From Fig. 5A it can be noted that the energy of the minima of RP-A, RP-B and RP-C sequentially increases, indicating that the electron transfer is energetically possible uphill. A recent study^19^ has shown that the relative energies decrease from RP-A through RP-C, when considering the presence of the environment and the thermal fluctuations of the protein, while in Fig. 5A the fluctuations of the protein have been neglected, which is the expected reason for the observed increase of relative radical pair energies.

To account for protein fluctuations, electronic spectrum calculations for several statistically independent configurations of the system should be carried out, yielding the statistics to evaluate the relative energies of the radical pair states more precisely. These configurations can, for example, be obtained from molecular dynamics simulations, and Fig. 5B shows the averaged energies for the key electronic states of *AtCry* active site, computed for independent configurations of the protein, taken as snapshots from earlier molecular dynamics simulations of the radical pair states^14^. The error bars are shown for the minima of the CS, RP-A, RP-B and RP-C states and arise due to the spread of energies. The error bars for the other states are not shown in order not to overcomplicate the plot. The calculated average energies and their standard deviations, shown in parenthesis, for the electronic states in the four configurations, are compiled in Table 2. A sampling of only 5 configurations, is likely not sufficient for achieving good statistics of the free energy, but is used here, as a proof of concept.

An important feature of Fig. 5B is the notable decrease in energy occurring due to the ET2 and ET3 processes, describing the 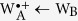 and 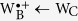 electron transitions. This is different in comparison to Fig. 5A, where increase in the driving force for these electron transfers was observed, and consistent with our earlier finding^19^.

Interestingly, that for some of the considered snapshots, the energies of the minima of the RP-B and RP-C states turned out to be lower than the energy of the CS state, as also suggested in Fig. 3. Such a behaviour favours radical pair stabilisation, as radical pair recombination becomes energetically unfavourable.

Embedding all components of the active site inside *AtCry* individually and averaging the excitation energies increases the energy of the RP-A_opt_ state as compared to the case where the entire active site, is embedded inside the protein, see Fig. 5A. Moreover, one notes that after the performed energy averaging, the energy of the RP-B_opt_ state turns out to be lower than the energy of the persistent RP-C_opt_ state. This is happening mainly due to a limited number of snapshot calculations, but is interesting since *AtCry* shows that at certain conditions the persistent radical pair is shifted from RP-C to RP-B, which is possible if the RP-B_opt_ state has lower energy than the RP-C_opt_ state, as was noted earlier^19^.

### Electron transfer rate constants

The computed energies of the various electronic states in *AtCry* could be used to estimate the rate constants for the different electron transfer reactions, i.e. ET2 and ET3. This can, for instance be achieved by using the Marcus theory of electron transfer^35^, which suggests


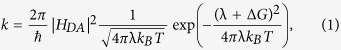


where 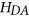 is the electronic coupling between the initial and final states of the donor and acceptor states participating in the electron transfer reaction, λ is the reorganization energy of the two states and 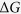 is the driving force of the electron transfer reaction, described as the energy difference between the minima of the free energy surfaces for the initial and final states. *T* is the temperature, and 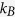 is the Boltzmann constant.

Within the framework of the Marcus theory, the electron transition from the donor to the acceptor parts of a molecular system is described by a generalized reaction coordinate, which relies on a coherent motion of all related nuclear and electronic degrees of freedom. Denoting all internal coordinates in the molecular system that participate in the electron transfer as a generalized reaction coordinate allows to represent the change of the energies of the electronic states arising upon electron transfer as two-dimensional energy profiles, shown in Fig. 5, even though the problem has a multidimensional nature.

The electronic coupling 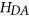 in Eq. 1 describes the coupling between the orbitals (wavefunctions) of the donor and acceptor states. Treating the donor and acceptor as two bound states separated by a barrier (the intermediate space between the donor and acceptor), i.e. describing the system with a square barrier tunneling model, leads to an asymptotic behaviour of 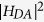 as an exponentially decreasing function^36^ with the distance *R*, the edge-to-edge distance between the tryptophans involved in the electron transfer,


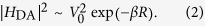


This assumption yields an approximate expression for the rate constant at 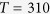 K in the following form


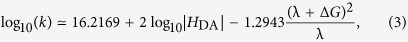


where one assumes λ ∼ 1 eV, 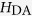, λ, and 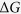 are counted in the units of eV. The value of 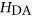 for the 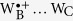 pair has been estimated previously as ~0.003 eV^19^. Here we use this number to estimate the rate constants for the ET2 and ET3 transfers. The values for the reorganization energy, distance between the donor and acceptor partners and the driving force, in Eq. (3) are compiled in Table 3 along with the calculated rate constants.

**Figure 2:**
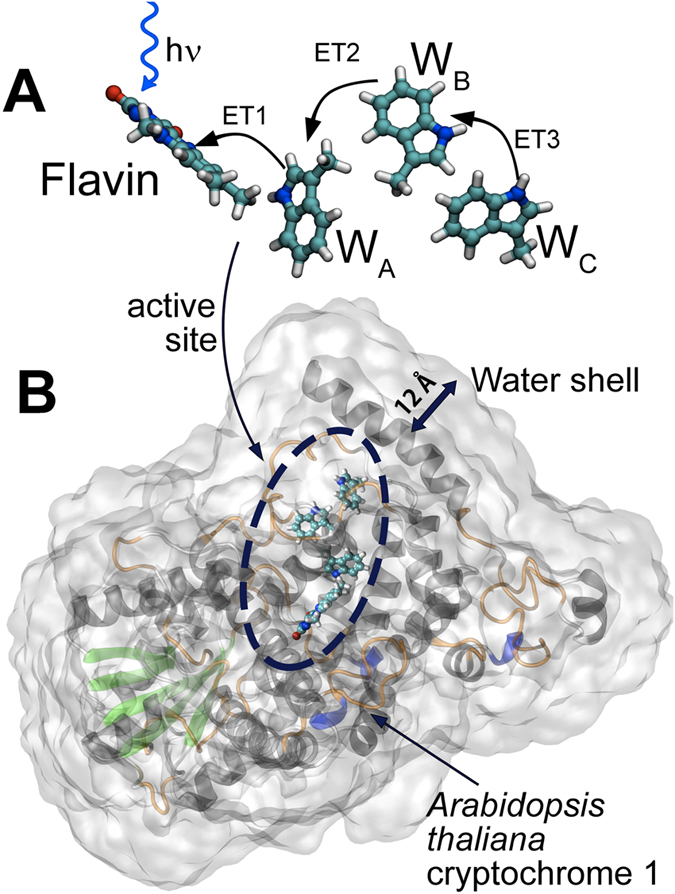
The tryptophan triad and the flavin cofactor constitute the active site of *AtCry*. The protein is activated once the flavin moiety has gained a radical character which is governed through three electron transfer steps, ET1, ET2 and ET3, between flavin and the tryptophan triad. The electron transfer ET1 is initiated by light excitation (**A**). Here we study these electron transfers for two different structural models of cryptochrome active site: (**A**) The *‘vacuum model’*, where only the active site is considered and all the protein interactions are neglected. The dangling bonds are terminated with the hydrogen atoms as shown. (**B**) The *‘environment model’*, where the complete protein structure and surrounding water shell are taken into account.

Experimental kinetics, obtained by Immeln *et al.*^37^ suggest that the electron transfer from 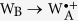 (ET2 in Fig. 2A) has a rate constant of 67–250 ns^−1^ and the electron transfer from 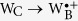 (ET3 in Fig. 2A) has a rate constant of 20–33 ns^−1^. The estimated rate constant for the ET2 electron transfers turn out to be in a reasonable agreement with the experimental values, however, the value for the ET3 rate constant is smaller than the measured one. The main major reasons for this is the sensitivity of the Marcus equation 1, to the values of 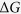, λ and 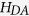. Indeed the electronic coupling parameter 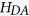 depend on 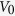 and *β*, see Eq. (2). Thus, if the distance between W_B_ and W_A_ changes by 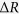, the coupling coefficient increases (if 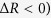 or decreases (if 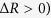 as


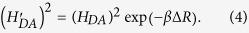


Assuming the generic value for 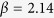 Å^−1^^10^ and 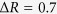 Å, the rate constant would decrease five-fold. Such a fluctuation of the interresidual distance is not to be unlikely, and in earlier MD simulations it was shown that fluctuations between tryptophan residues in *AtCry* of about 1 Å are easily possible^14^. Earlier QM/MM simulations^19^ also demonstrate independently that increasing the distance between electron donor and acceptor by ~0.5 Å leads to a three-fold decrease of the coupling coefficient 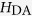 lowering the electron transfer rate. Using a large amount of snapshots, to get better statistics for the free energy surface, could alleviate the uncertainty, however, the computational effort would be immense.

## Conclusion

Quantum biology has developed over the past decade as a result of convergence between quantum physics and biology. This emerging field stems from the interrogation of the basic principles that govern interactions at the molecular scale in living organisms. New experimental techniques have provided evidence that phenomena such as photosynthesis, birds’ orientation in the Earth’s magnetic field, smell and possibly anaesthesia may be due to quantum effects^38^.

Electron transfer reactions involve the movement of an electron from one molecular species (the donor) to another (the acceptor) and turn out to be an essential quantum mechanical component in various biological processes. In this paper we considered electron transfer from a theoretical view-point; namely in terms of quantum mechanical and semi-classical models. Specifically we describe the electron transfer through the time-dependent DFT formalism, employ the diabatic representation of electronic states along a reaction coordinate and finally utilize Marcus theory to estimate the electron transfer rate constants.

In the present investigation of electron transfer reactions in biological systems we concentrate on a specific example of plant cryptochrome (*AtCry*), where electron transfers are essential for functioning^12^. Inspired by a previous investigation^19^ we seek to establish the physical role of different interactions in the system, and to deduce those that play the major role in electron transfer reactions. For that purpose we employ the polarizable embedding approach, where, the quantum mechanical region of interest consists of the active site of *AtCry*, while the classical region consists of the remaining part of the system.

We have discovered that the electron transfer reactions in *AtCry* are stabilised significantly by the protein, and that the electron transfer reaction cannot be modelled accurately by considering the active site in isolation. We have established that electrostatics is crucial in the electron transfer reactions, and furthermore we have deduced that, in addition to the static multipoles (charges, dipoles and quadrupoles), the polarisation forces play the key role, in propelling the electron through cryptohrome. Lastly, the present investigation revealed that thermal fluctuations of the protein are crucial, in order to obtain accurate estimates for the energy surfaces of the key electronic states involved in the electron transfer reactions; neglecting the fluctuations could lead to potential energy surfaces that are energetically uphill, making electron transfer less favourable.

The quantum mechanical description of electron transfer reactions employed in this work is general and applicable to a variety of biological systems, such as, for example, DNA photolyase^2^ or the bc1-complex^7^, were electron transfer occurs in well defined parts of the systems. The applications of the polarisable embedding method to electron transfer dynamics is especially attractive since the method is free of model parameters and captures all important physics and chemistry in a complex molecular system. The method provides a robust possibility to describe quantum dynamics with account for the entire molecular system and can easily be extended to take account of the thermal fluctuations. In the view of an enormous number of model and *ab initio* approaches available nowadays, we would, thus, like to conclude that polarizable embedding approach is far among the promising tools for challenging various problems in quantum biology in the nearest future.

## Methods

In this section we discuss the computational methods that were used to describe the electron transfer reactions in *AtCry*. First we describe the computation of the electronic properties of the *AtCry* active site *in vacuo*. Then we introduce the polarizable embedding method, that was used to account for interactions of the active site with the remainder of cryptochrome. In the supplementary information, we outline the simulation protocol employed.

### Structural optimization of the radical pair states in vacuum

**Figure 3:**
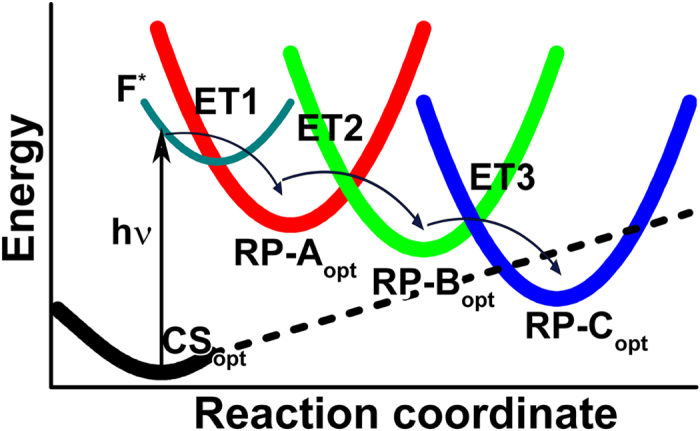
Schematic depiction of the free energy surfaces for the five key electronic states in *AtCry*. The free energy surface of the CS state is shown in black, for the radical pair RP-A state in red, the RP-B state in green and the RP-C state in blue. Thin cyan line shows the free energy surface of the *AtCry* with flavin photoexcited. Crossing of the free energy surfaces renders the electron transfer between the two corresponding states possible, which are depicted as ET1, ET2, ET3. The minima on the free energy surfaces, denoted CS_opt_, RP-A_opt_, RP-B_opt_, RP-C_opt_, correspond to the optimized structural configurations of *AtCry* active site in either the CS, RP-A, RP-B or RP-C states respectively.

**Figure 4:**
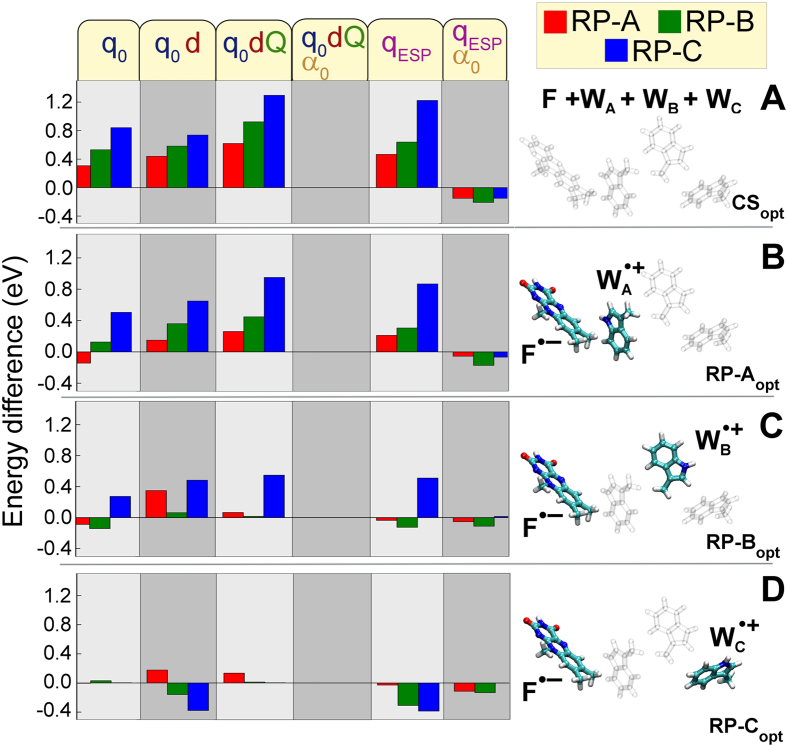
Energetic differences between the individual interactions and *q*_0_, *d*, *Q*, *α*_0_, *α*_1_. The energy differences for the four structural configurations of the cryptochrome active site, with each of the different set of interactions (top labels), are computed relative to the energies where all interaction from the environment (*q*_0_, *d*, *Q*, *α*_0_, *α*_1_) are included, see Fig. S2. (**A**) Relative energies for the closed shell configuration, CS_opt_, (**B**) the radical pair RP-A_opt_ configuration, (**C**) the radical pair RP-B_opt_ configuration and (**D**) the radical pair RP-C_opt_ configuration. Each panel shows the energy differences computed if only some interactions of the active site and environment are considered (labels top row). For example the first column shows the difference between the interactions *q*_0_ and *q*_0_, *d*, *Q*, *α*_0_, *α*_1_, i.e. 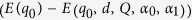. Color indicates the different electronic states, RP-A, RP-B and RP-C, for a given structural configuration of the active site (shown in the right part of the figure): radical pair state RP-A (red), radical pair state RP-B (green) and for the radical pair state RP-C (blue).

**Figure 5:**
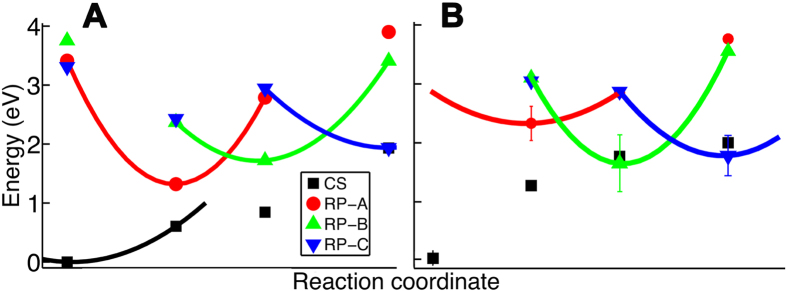
Schematic depiction of the energy profiles of the four electronic states in *AtCry*. The energy for the oxidized flavin state is shown in black, for the radical pair RP-A state in red, RP-B state in green and RP-C in blue. The symbols represent the calculated energies and the lines are schematic representations of the energy surfaces. (**A**) Shown are the potential energies computed for the optimized vacuum models of the CS, RP-A, RP-B and RP-C states, embedded inside *AtCry*. Here the four states CS, RP-A, RP-B and RP-C, are studied with account for the protein environment and include electrostatic and polarization interactions between the active site and the environment. (**B**) Average energies, representing the free energy, computed from snapshot calculations, where the isoalloxazine moiety of the FAD, the three tryptophans of the *AtCry* active site were embedded inside *AtCry* individually, as described in Methods. The environment embedding potential in this case is considered including 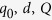 order of multipole expansion and the polarization term governed by 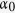, being physically equivalent to 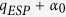. Each point is the mean value of 5 calculations.

The cryptochrome active site, shown in Fig. 2A, and the environment model of the protein, shown in Fig. 2B were constructed from the *AtCry* crystal structure (PDB code 1U3C)^22^. Geometry optimization of the active site (vacuum) models involved its different redox states characterized through (i) the oxidized flavin, FAD, with neutral (reduced) W_A_, W_B_ and W_C_ residues, (ii) the radical pair 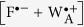 with reduced W_B_ and W_C_ (iii) the radical pair 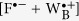 with reduced W_C_ and W_A_, and (iiii) the radical pair 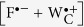 with reduced W_B_ and W_A_. The optimizations were performed with the state-averaged CASSCF^39^ method employing the protocol developed earlier^40^, assuming equal weights for the states considered; the LUMO of the flavin and the HOMOs of W_C_, W_B_ and W_A_ were included in the CASSCF active space, during the optimization the 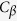 were constrained. The CASSCF wavefunctions were selected according to the principal-orbital complete active space approach^14^,^16^,^41^, where the single-electron excitations, corresponding to the radical pair states are described by including two molecular orbitals in the CASSCF active space. At the optimized geometries (see minima denoted with subscript ‘opt’ in Fig. 3), the excitation spectra were computed using the CASSCF^39^, XMCQDPT2^42^ and KS-DFT using the CAM-B3LYP exchange-correlation functional^43^,^44^. The electronic wavefunction was expanded in all calculations using the 6-31G* basis set.

### Polarizable embedding approach

The polarizable embedding (PE) method^45^,^46^ is a combined QM/MM-type scheme that focuses on the prediction of molecular response properties using polarizable embedding potentials derived from quantum-mechanical calculations. It has been formulated for Hartree-Fock (HF)^45^, multi-configurational self-consistent field (MCSCF)^47^ and coupled cluster (CC)^48^,^49^ wave functions as well as Kohn-Sham density functional theory (KS-DFT)^45^, including in all cases also time-dependent response theory^50^. The PE method is an efficient and accurate computational scheme for large molecular structures where the property of interest is localized in specific parts of the system. The molecular system is thus divided into a **core region**, described at a quantum mechanical level of theory, and the **environment region**, which is included as an embedding potential that enters the Hamiltonian of the core region. In the present investigation the core region is defined as the active site of *AtCry*, while the environment is represented by the rest of the system (protein matrix and surrounding water molecules and ions).

The embedding potential consists of atom-centered permanent multipole moments, i.e. charges, dipoles, quadrupoles etc. The permanent multipoles are used to model the interactions between the active site and the static charge distribution of the environment. In addition, atom-centered dipole-dipole polarizabilities are used to allow mutual polarization between the core and environment regions. The polarizabilities give rise to induced dipoles which describe the induced charge distribution of the environment in the presence of electric fields. The induced dipoles depend on the fields from the active site electrons and nuclei, and from the permanent and induced multipoles in the environment. Since the induced dipoles depend on the electronic state of the active site via the electric field, they are updated in each step during the optimization of the core-region ground-state density/wave function. In addition, the induced dipoles are also determined self-consistently in the calculation of properties and excitation energies using a response theory formalism.

### Electronic structure calculations of *AtCry* with account of environment effects

To quantify which interactions from the environment have the largest impact on the electron transfer in *AtCry*, we have used four structures of *AtCry* active site, optimized in the four electronic states, CS, RP-A, RP-B, RP-C in vacuum (see description above). To describe the geometry of the active site in a realistic environment, we have then superimposed the optimized vacuum models obtained, with the rest of the protein in water. The new structures were then equilibrated, using the NAMD package^51^, for 10 ns employing the CHARMM36 force field^52^,^53^. A time step of 2 fs was used and the temperature was controlled at 310 K using the Langevin thermostat. Similarly the pressure was held at 1 atm with the Langevin Barostat^54^. The ShakeH algorithm was used to keep bonds involving hydrogen atoms at fixed lengths. During the equilibration the atoms of the active site were constrained, as to preserve the optimized structures of the different electronic states obtained earlier. The equilibrated structures were then used in the PE calculations, where the active site of cryptochrome was treated quantum mechanically including the effect of the environment via an embedding potential. For these calculations we employed the Dalton program^55^, http://daltonprogram.org, utilizing the PE library^56^ and Gen1Int^57^,^58^, using the KS-DFT method with the CAM-B3LYP exchange-correlation functional and the 6-31G* basis set. The embedding potential parameters are derived by dividing the molecular system surrounding the active site into smaller fragments using the molecular fractionation with conjugate caps (MFCC) approach^59^. The distributed multipole moments and polarizabilities are then calculated for each fragment separately and the final parameters are assembled using the MFCC principle as formulated by Söderhjelm and Ryde^60^. Calculations of the potential were carried out using KS-DFT with the B3LYP exchange-correlation functional and the 6–31+G* basis set, with an in-house script, developed by one of the authors. Embedding potentials of this kind have previously been shown to be very accurate^33^.

The four static calculations of the electronic states, CS, RP-A, RP-B, RP-C with account for the environment, are, however, not sufficient to describe the dynamical changes within the protein. The electronic structure of the CS, RP-A, RP-B, RP-C states has, therefore, been explored further using the PE formalism for several independent configurations of the protein, taken from snapshots of earlier molecular dynamics simulations^14^. In particular we have used 5 snapshots from molecular dynamics simulations of *AtCry* in each of the CS, RP-A, RP-B, and RP-C states. For each snapshot we have superimposed the individual components of the active site (isoalloxazine moiety of the FAD, the three tryptophans) from the optimized vacuum models of the corresponding electronic state with the rest of the protein in water. This is done by first superimposing only the FAD part of the vacuum model, with the FAD part of the protein, and second superimposing only the W_A_ from the active site with the W_*A*_ from the protein, etc. This allows the tryptophans and the FAD to change orientation with respect to each other, to account for structural changes in the protein. The new structures were then equilibrated for 1 ns and prepared for the PE calculations, employing the protocol outlined above.

## Additional Information

**How to cite this article**: Sjulstok, E. *et al.* Quantifying electron transfer reactions in biological systems: what interactions play the major role? *Sci. Rep.* **5**, 18446; doi: 10.1038/srep18446 (2015).

## Supplementary Material

Supplementary Information

## Figures and Tables

**Figure 1:**
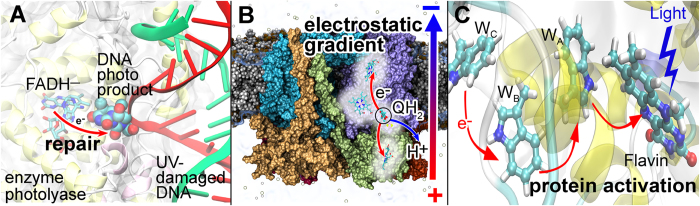
Examples of biological systems where electron transfer play a key role. (**A**) electron transfer initiating DNA UV-lesion repair by enzyme photolyase. (**B**) electron transfer triggering a cascade of charge transfer reactions in the cytochrome bc1 complex that lead to a formation of an electrostatic gradient through the plasma membrane. (**C**) Activation of cryptochrome protein initiated by blue light excitation of the FAD cofactor leading to a formation of a radical pair.

**Table 1:**
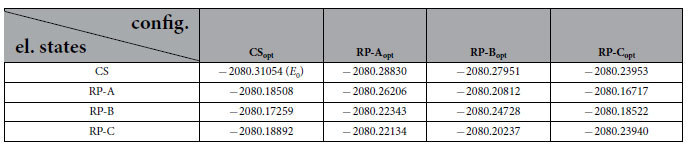
Calculated total energies (in a.u.) of the four electronic states (closed shell, RP-A, RP-B and RP-C), considered in the four optimized configurations CS_opt_, RP-A_opt_, RP-B_opt_ and RP-C_opt_ see Fig. 3.

The energies were used to compute the energy diagrams shown in Fig. 5A.

**Table 2:**
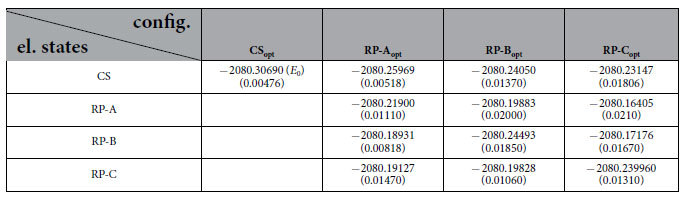
Total averaged energies (in a.u.) of the four electronic states (closed shell, RP-A, RP-B and RP-C) computed for the four optimized configurations CS_opt_, RP-A_opt_, RP-B_opt_ and RP-C_opt_ of *AtCry* active site.

The averaging has been performed over 5 independent calculations for configurations taken from an earlier MD simulation, as described in Methods. The values in the brackets indicate energy standard deviations for each state/configuration. The energies were used to compute the energy diagrams shown in Fig. 5B.

**Table 3:**
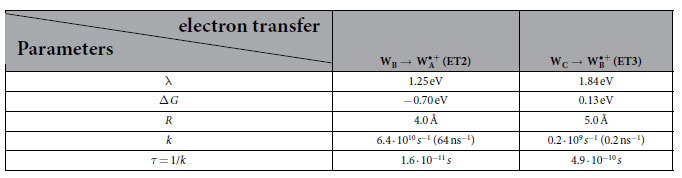
Parameters used in Eq. (3), derived from Fig. 5B and the equilibrated structure of *AtCry*, along with the calculated rate constants, for the two electron transfers, ET2 and ET3, labeled in Fig. 2.
