# Supplementary material for: Quantifying electron transfer reactions in biological systems: what interactions play the major role?
Source: Sci Rep. 2015 Dec 22;5:18446. doi: 10.1038/srep18446 (PMC4686879; doi:10.1038/srep18446)
Supplement: Supplementary Information [file srep18446-s1.pdf]

Quantifying electron transfer reactions in biological systems: what interactions play the major role? (Supplementary information)

Emil Sjulstok[⋆,†], Jógvan Magnus Haugaard Olsen[⋄⋆] & Ilia A. Solov'yov[⋆,‡]

November 2, 2015

⋆ Department of Physics, Chemistry and Pharmacy, University of Southern Denmark, DK-5230 Odense M, Denmark
⋄ Laboratory of Computational Chemistry and Biochemistry, École Polytechnique Fédérale de Lausanne (EPFL), CH-1015 Lausanne, Switzerland
† Email: emilsr@sdu.dk
‡ Email: ilia@sdu.dk

Methods

Simulation protocol

The hybrid quantum mechanics/molecular mechanic (QM/MM) calculations with the PE approach are rather involved, as they include many technical steps, necessary to perform the calculations accurately. Below we summarize the crucial steps that are needed for such calculations. Each calculation constitutes four steps:

1. Fragmenting the molecular system into the core and the environment regions.

2. Obtaining the embedding potential for the environment region.

3. Handling the molecular interface between the core and the environment regions.

4. Calculating the desired properties of the core region.

First the protein is subdivided into two regions, the core region, or the active site, and the environment (here we denote the molecular subsystem outside the active site as the environment), by using the dedicated VMD[1] extension pe-fragmenter (available in house). During this procedure, covalent bonds connecting the active site and the environment are broken, and the resulting dangling bonds are then capped with hydrogen atoms. In the present study, the active site consists of the side chains of the three tryptophans and the isoalloxazine moiety of the FAD, as shown in Fig. 2A. The environment includes the remainder of the protein and a shell of water molecules and ions surrounding the protein, with a layer of 12 Å, see Fig. 2B

Second, the embedding potential is calculated using an in-house script, developed by one of the authors. Here all residues (amino acids) of the environment are treated as overlapping fragments, according the MFCC approach, and the potential is represented through electrostatic components of the individual residues. For *AtCry* the protein is divided into

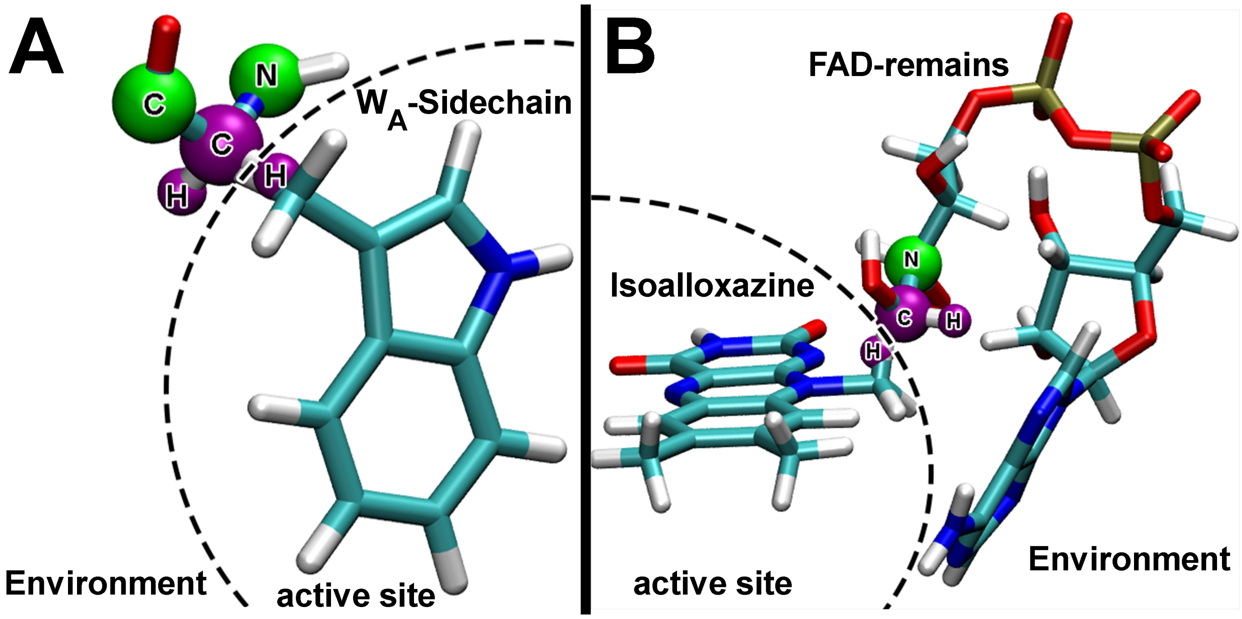

Figure S1: **Computational description of the interface between the core and environment regions.** Graphical depiction of an overlap between the active site and the environment, due to breaking of covalent bonds in the course of the PE calculations and termination of the dangling bonds with hydrogens. **A**) between W_A backbone and its sidechain, **B**) between the isoalloxazine moiety and FAD remains. In both cases, the charges of the atoms marked purple are redistributed to the atoms marked green.

969 fragments. Next, the interface between the core and environment subsystems should be taken care of. Capping the cut covalent bonds between the active site and the environment leads to an near-overlap of atoms from the two regions, as for example illustrated in Fig. S1. This overlap is handled by redistributing the charges from the atoms that clash with the active site, to the two nearest heavy atoms, equally, to the two nearest heavy atoms, i.e. non-hydrogen atoms. Figure S1 shows examples of such redistributions in the case of tryptophan and isoalloxazine cofactor. The remaining contributions to the embedding potential, i.e. higher-order multipole moments and polarizabilities for the conflicting atoms are deleted. Finally, the properties of the active site in the embedding potential are calculated using quantum chemistry software, e.g. Dalton[2,3].

Results

Environment stabilizes radical pair states.

The electronic excitation spectra of the *AtCry* active site are studied for the four optimized structural configurations of the active site, namely, [F+ W_A+W_B+W_C] ≡ CS; [$F^{\bullet-}$+ $W_A^{\bullet+}$+W_B+W_C] ≡ RP-A; [$F^{\bullet-}$+W_A+ $W_B^{\bullet+}$ + W_C] ≡ RP-B and the final, persistent, radical pair state [$F^{\bullet-}$+W_A+W_B+$W_C^{\bullet+}$] ≡ RP-C, introduced earlier. These four optimised configurations correspond to the minima CS_{opt}, RP-A_{opt}, RP-B_{opt}, RP-C_{opt} shown in Fig. 3.

Figure S2 shows the calculated spectra, obtained for the four structural configurations. Here symbols represent energies of certain electronic states; in the case of the environment model (Fig. 2B), with all five components of the electrostatic interactions and polarization $(q_0, d, Q, \alpha_0, \alpha_1)$ included, the spectra are labeled as $q_0 dQ\alpha_0\alpha_1$. The excitation energies in Fig. S2 are shown relative to the energy of the CS state for the given structure, shown as black squares.

The excitation energies calculated for the vacuum models of the active site (Fig. 2A), are highlighted in Fig. S2 through the blue background, and are shown in the left part of the figure. The comparison of the corresponding energy spectra calculated for the two models (vacuum computed with CAM-B3LYP vs. environment), shows that accounting for the environment lowers the energy of the excited states for all structural configurations: for the configurations RP-A_{opt}, RP-B_{opt}, RP-C_{opt} the energy is lowered by ∼1.8 eV (and more), while for the closed shell configuration CS_{opt} the energy is lowered by 0.4 eV, i.e. the effect of the environment is less pronounced, but still significant.

Note that the presence of a molecular environment preferentially stabilizes the electronic state, for which the structure of the active site was optimized. Indeed, the RP-A excited state (red dot) for the RP-A_{opt} (see Fig. 3) structure of *AtCry* gets a more profound decrease in energy compared to the RP-B and RP-C states, lastly RP-B excited state (green triangle)

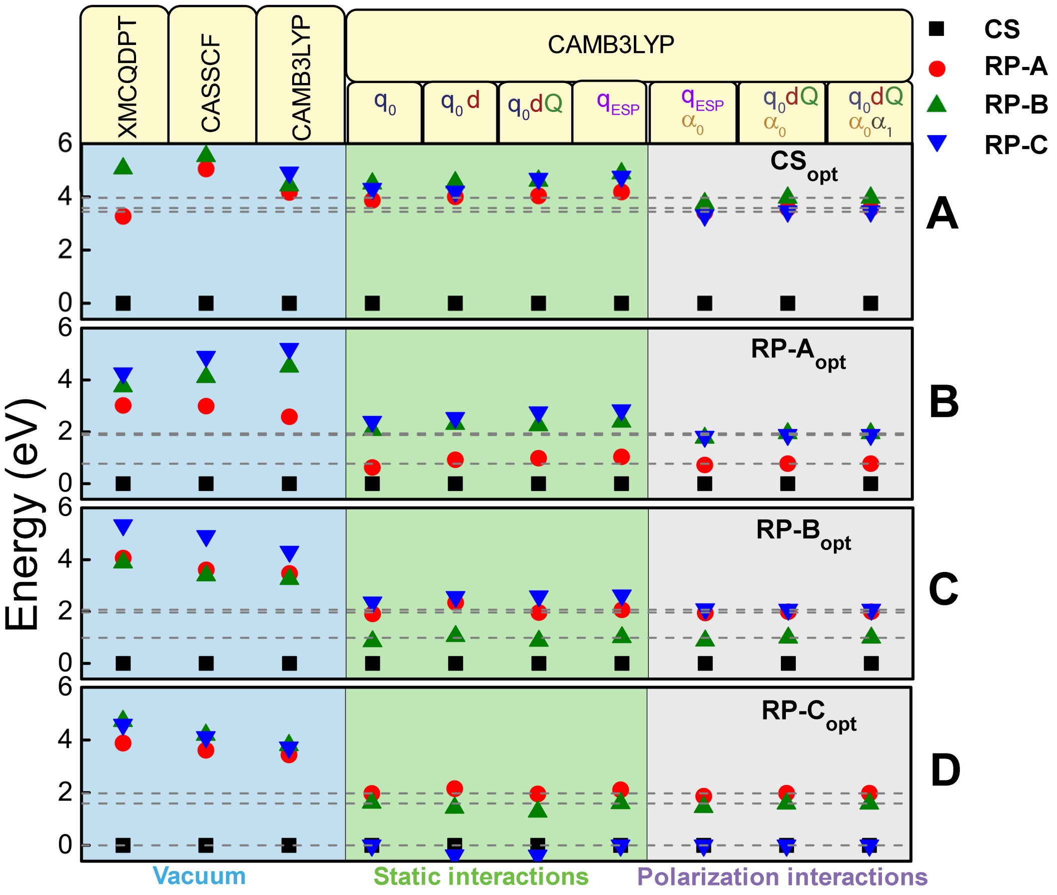

Figure S2: **Impact of different interactions on the energies for the key electronic states in *AtCry*.** For both models of the active site (see Fig. 2), four structural configurations are considered: **A:** the closed shell, CS_{opt} **B:** the radical pair $RP\text{-}A_{opt}$, **C:** the radical pair $RP\text{-}B_{opt}$ and **D:** the radical pair $RP\text{-}C_{opt}$. For all the configurations the geometry of the active site was optimized for the vacuum model, and the obtained structures were then used for the environment model, as described in the text. For each of the structural configurations four electronic states are studied, associated with the different redox states of the active site at a given geometry: the oxidized flavin state (black squares), the radical pair state RP-A (red dots), the radical pair state RP-C (green upper triangles) and the radical pair state RP-C (blue lower triangles). Calculations of the electronic states in the vacuum model have been carried out using the XMCQDPT-2, CASSCF and DFT-CAMB3LYP methods. The environment model was studied using the DFT-CAMB3LYP approach only. Here the interactions of the active site with the molecular environment have been decomposed into five contributions, representing the environment through: partial charges (q_0), dipole moments (d) and quadrupole moments (Q) ascribed to each atom of the protein; point charges (q_{ESP}) fitted to reproduce the electrostatic potential; induced dipole moments on each atom of the environment, calculated from the ground state of the active site (α_0); induced dipole moments of all atoms of the environment, that take into account also the charge redistribution in the system upon electronic excitation (α_1).

is more stabilized in the case of the RP-B_{opt} structure, while RP-C stabilization leads to a dramatic energy decrease in the case of RP-C_{opt} structure. This stabilization is absent in the vacuum model, however, it is of significant importance since it indicates that environment leads to the energetic favoring of a certain electronic state.

Electrostatic interactions and polarization capture the physics of electron transfer.

As shown above, the environment of the active site can be represented through the ESP-fitted charges and ground state polarization (q_{ESP}+α_0), to a fairly good approximation. One benefit of using this approach is, that it permits modelling molecular environment in a variety of quantum chemistry programs, such as Dalton[2,3], Gaussian[4], MOLCAS[5,6], and Firefly[7], whereas the multipole expansion model, though giving slightly better results, is not widely implemented, and can presently only be used with Dalton via the PE-library[8]. Different quantum chemistry programs have, sometimes, different functionalities, and, therefore, allow calculating different properties of the active site.

Moreover, using the multipole expansion approach with Dalton[2,3] does not allow for open-shell singlet diradical DFT calculations, which can become problematic if the closed shell state and one of the radical pair states become close in energy. Such a situation is, for example, possible in *AtCry* under certain conditions as demonstrated below.

Electronic properties of *AtCry* could be modeled with CAM-B3LYP.

The calculations for the environment model are carried out with TDDFT method using the CAM-B3LYP exchange-correlation functional, as described in Methods. To examine whether TDDFT can adequately describe the electron transfers in *AtCry* we have considered the electronic excitation spectra of its active site, optimized in four structural configurations in

vacuum (the CS_{opt} state, the radical pair states RP-A_{opt}, RP-B_{opt} and RP-C_{opt}, see Fig. 2A and Fig. 3). For the optimized configurations we then have computed additionally the electronic excitation spectra by using the XMCQDPT-2[9], CAM-B3LYP[10–13] and CASSCF[14,15] methods. Figure S2 (left part) summarizes the comparison between the different calculations.

It can be seen that for all the structural configurations of the active site, the order of electronic excitations is the same for the three methods, indicating that overall CAM-B3LYP yields qualitatively correct results. For all structural configurations, except the RP-A_{opt}, the energy of all radical pair states computed with CAM-B3LYP is lowered, as compared to the energies calculated with XMCQDPT-2 and CASSCF methods. It should be noted that CASSCF is known to overestimate the energy of excited states[16], which accounts for some of the discrepancies between the CASSCF and CAM-B3LYP approaches.

References

1. Humphrey, W., Dalke, A., and Schulten, K. VMD – visual molecular dynamics. *J. Molec. Graphics* **14**, 33–38 (1996).

2. Aidas, K., Angeli, C., Bak, K. L., Bakken, V., Bast, R., Boman, L., Christiansen, O., Cimiraglia, R., Coriani, S., Dahle, P., Dalskov, E. K., Ekström, U., Enevoldsen, T., Eriksen, J. J., Ettenhuber, P., Fernández, B., Ferrighi, L., Fliegl, H., Frediani, L., Hald, K., Halkier, A., Hättig, C., Heiberg, H., Helgaker, T., Hennum, A. C., Hettema, H., Hjertenæs, E., Høst, S., Høyvik, I.-M., Iozzi, M. F., Jansík, B., Jensen, H. J. A., Jonsson, D., Jørgensen, P., Kauczor, J., Kirpekar, S., Kjærgaard, T., Klopper, W., Knecht, S., Kobayashi, R., Koch, H., Kongsted, J., Krapp, A., Kristensen, K., Ligabue, A., Lutnæs, O. B., Melo, J. I., Mikkelsen, K. V., Myhre, R. H., Neiss, C., Nielsen, C. B., Norman, P., Olsen, J., Olsen, J. M. H., Osted, A., Packer, M. J., Pawlowski, F., Pedersen, T. B., Provasi, P. F., Reine, S., Rinkevicius, Z., Ruden, T. A., Ruud, K., Rybkin, V. V., Sałek, P., Samson, C. C. M., de Merás, A. S., Saue, T., Sauer, S. P. A., Schimmelpfennig, B., Sneskov, K., Steindal, A. H., Sylvester-Hvid, K. O., Taylor, P. R., Teale, A. M., Tellgren, E. I., Tew, D. P., Thorvaldsen, A. J., Thøgersen, L., Vahtras, O., Watson, M. A., Wilson, D. J. D., Ziolkowski, M., and Ågren, H. The dalton quantum chemistry program system. *Wiley Interdisciplinary Reviews: Computational Molecular Science* **4**, 269–284 (2014).

3. Dalton, a molecular electronic structure program, Release Dalton2013.4, (2013). http://daltonprogram.org.

4. Frisch, M. J., Trucks, G. W., Schlegel, H. B., Scuseria, G. E., Robb, M. A., Cheeseman, J. R., Scalmani, G., Barone, V., Mennucci, B., Petersson, G. A., Nakatsuji, H., Caricato, M., Li, X., Hratchian, H. P., Izmaylov, A. F., Bloino, J., Zheng, G., Sonnenberg, J. L.,

Hada, M., Ehara, M., Toyota, K., Fukuda, R., Hasegawa, J., Ishida, M., Nakajima, T., Honda, Y., Kitao, O., Nakai, H., Vreven, T., Montgomery, Jr., J. A., Peralta, J. E., Ogliaro, F., Bearpark, M., Heyd, J. J., Brothers, E., Kudin, K. N., Staroverov, V. N., Kobayashi, R., Normand, J., Raghavachari, K., Rendell, A., Burant, J. C., Iyengar, S. S., Tomasi, J., Cossi, M., Rega, N., Millam, J. M., Klene, M., Knox, J. E., Cross, J. B., Bakken, V., Adamo, C., Jaramillo, J., Gomperts, R., Stratmann, R. E., Yazyev, O., Austin, A. J., Cammi, R., Pomelli, C., Ochterski, J. W., Martin, R. L., Morokuma, K., Zakrzewski, V. G., Voth, G. A., Salvador, P., Dannenberg, J. J., Dapprich, S., Daniels, A. D., Farkas, Ö., Foresman, J. B., Ortiz, J. V., Cioslowski, J., and Fox, D. J. Gaussian 09 revision d.01, (2009). Gaussian Inc. Wallingford CT 2009.

5. Aquilante, F., Vico, L. D., Ferré, N., Ghigo, G., åke Malmqvist, P., Neogrády, P., Pedersen, T. B., Pitoňák, M., Reiher, M., Roos, B. O., Serrano-Andrés, L., Urban, M., Veryazov, V., and Lindh, R. MOLCAS 7: the next generation. *J. Comp. Chem.* **31**, 224–247 (2010).

6. Veryazov, V., Widmark, P.-O., Serrano-Andrés, L., Lindh, R., and Roos, B. O. 2molcas as a development platform for quantum chemistry software. *Int. J. Quantum Chem.* **100**, 626–635 (2004).

7. Granovsky, A. A. Firefly version 8.0.0. http://classic.chem.msu.su/gran/firefly/index.html, (2015).

8. Olsen, J. M. H. PElib: The Polarizable Embedding library (version 1.0.8), (2014).

9. Granovsky, A. A. Extended multi-configuration quasi-degenerate perturbation theory: The new approach to multi-state multi-reference perturbation theory. *J. Chem. Phys.* **134**, 214113–(1–14) (2011).

10. Stephens, P. J., Devlin, F. J., Chabalowski, C. F., and Frisch, M. J. Ab initio calculation of vibrational absorption and circular dichroism spectra using density functional force fields. *J. Phys. Chem.* **98**, 11623–11627 (1994).

11. Lee, C., Yang, W., and Parr, R. G. Development of the colle-salvetti correlation-energy formula into a functional of the electron density. *Phys. Rev.* **37**(2), 785–789 (1988).

12. Becke, A. D. Density-functional thermochemistry. III. the role of exact exchange. *J. Chem. Phys.* **98**(7), 5648–5652 (1993).

13. Yanai, T., Tew, D., and Handy, N. A new hybrid exchange-correlation functional using the coulomb-attenuating method (CAM-B3LYP). *Chem. Phys. Lett.* **393**, 51–57 (2004).

14. Roos, B. O., Taylor, P. R., and Siegbahn, P. E. A complete active space SCF method (CASSCF) using a density matrix formulated super-CI approach. *Chem. Phys.* **48**, 157–173 (1980).

15. Siegbahn, P. E. M., Almlöf, J., Heiberg, A., and Roos, B. O. The complete active space SCF (CASSCF) method in a Newton-Raphson formulation with application to the HNO molecule. *J. Chem. Phys.* **74**, 2384–2396 (1981).

16. Sala, M., Kirkby, O. M., Guérin, S., and Fielding, H. H. New insight into the potential energy landscape and relaxation pathways of photoexcited aniline from CASSCF and XMCQDPT2 electronic structure calculations. *Phys. Chem. Chem. Phys.* **16**, 3122–3133 (2014).
